# Use of Telemedicine in Depression Care by Physicians: Scoping Review

**DOI:** 10.2196/29159

**Published:** 2021-07-26

**Authors:** Jean-François Echelard

**Affiliations:** 1 Faculty of Medicine Université de Montréal Montreal, QC Canada

**Keywords:** telemedicine, telepsychiatry, depression, mental health, videoconferencing

## Abstract

**Background:**

Depression is a common disorder, and it creates burdens on people’s mental and physical health as well as societal costs. Although traditional in-person consultations are the usual mode of caring for patients with depression, telemedicine may be well suited to psychiatric assessment and management. Telepsychiatry can be defined as the use of information and communication technologies such as videoconferencing and telephone calls for the care of psychopathologies.

**Objective:**

This review aims to evaluate the extent and nature of the existing literature on the use of telemedicine for the care of depression by physicians. This review also aims to examine the effects and perceptions regarding this virtual care and determine how it compares to traditional in-person care.

**Methods:**

The Arksey and O’Malley framework and the PRISMA-ScR (Preferred Reporting Items for Systematic Reviews and Meta-Analyses Extension for Scoping Reviews) guidelines were followed. Relevant articles were identified through a search of three databases (MEDLINE, Cochrane Database of Systematic Reviews, and PsycArticles) on October 11, 2020. The search terms were “(*virtual* OR *telemedicine* OR teleconsultation* OR *telehealth* OR phone* OR webcam* OR *telepsychiatry*) AND (depress*)”. Eligibility criteria were applied to select studies about the use of telemedicine for the care of patients with depression specifically by physicians. An Excel file (Microsoft Corporation) was used to chart data from all included articles.

**Results:**

The search resulted in the identification of 28 articles, and all 13 nonreview studies were analyzed in detail. Most nonreview studies were conducted in the United States during the last decade. Most telemedicine programs were led by psychiatrists, and the average study population size was 135. In all applicable studies, telepsychiatry tended to perform at least as well as in-person care regarding improvement in depression severity, patient satisfaction, quality of life, functioning, cost-effectiveness, and most other perceptions and variables. Cultural sensitivity and collaborative care were part of the design of some telemedicine programs.

**Conclusions:**

Additional randomized, high-quality studies are recommended to evaluate various outcomes of the use of telemedicine for depression care, including depression variables, perceptions, health care outcomes and other outcomes. Studies should be conducted in various clinical contexts, including primary care. Telepsychiatry is a promising modality of care for patients suffering from depression.

## Introduction

### The Burdens of Depression

Depression is a very common disorder and thus represents a heavy mental health burden. Although estimates vary widely, the lifetime and 12-month prevalence of depression may be close to 10% and 5%, respectively, and these values are higher in high-income countries [1]. Suicidal risk and ideations have a high incidence among patients with depression. Although estimates vary widely, the incidence of suicide among patients with depression may be as high as 15%, while up to 70% of patients with acute depression may experience suicidal ideas [2]. Unfortunately, depression is associated with an elevated risk of early death. This is partly due to suicidal risk but also to the heavy physical health burden of this psychopathology. Indeed, depression is a predictor of many physical diseases, including coronary artery disease, myocardial infarction, stroke, diabetes, and various cancers. Certain physical disorders also tend to be more severe among patients with depression. There is a reciprocal relationship between depression and physical pathologies. Biologically plausible mechanisms include behaviors such as smoking, drinking alcohol, obesity, low compliance with treatment regimens, and a variety of hormonal and immune dysregulations. Unsurprisingly, depression is also associated with lower perceived overall health [1]. Caring for mentally and often physically sick patients is expensive and requires resources. Thus, the health care burden of depression is heavy. The costs of this disease have been increasing in recent decades and amount to hundreds of billions of dollars in the United States [3]. Depression is highly socially burdensome in several other ways. It is associated with termination of education, lower probability of marrying, marital dissatisfaction, negative parenting behaviors, adolescent childbearing, unemployment, work disability, absenteeism, low work performance, lower personal earnings, and lower household income [1]. Approximately half of all costs associated with depression are workplace costs [3].

### Approaches to Depression Care

Although traditional in-person consultations are the usual modality of caring for patients with depression, telemedicine may be well suited to psychiatric assessment and management. This modality of care may also offer many advantages, such as improving access to care in rural areas [4]. In fact, medicine as a whole is becoming increasingly digitalized as competencies required to provide health care are evolving [5]. Other components of depression care include collaborative care and culturally sensitive approaches. Collaborative care is an integrated model in which many health care professionals work together with the patient to better manage the patient’s disease in a synergic manner [6]. Cultural aspects must also be considered, as beliefs regarding depression and mood influence how patients perceive their psychological health [7].

### Defining Telepsychiatry

For the scope of this review, telepsychiatry is defined as the use of information and communication technologies such as videoconferencing and telephoning for the diagnosis and management of psychopathologies. Telepsychiatry is thus a synonym of telemedicine for mental health. Psychiatrists, general practitioners, and other physicians can practice telepsychiatry. Other health care professionals, including psychologists, social workers, and nurses, can use telemedicine for mental health as well. Broader or narrower definitions of telepsychiatry could be used by other authors. Various technologies could also be included in other definitions, including artificial intelligence, augmented or virtual reality, mobile apps, and the Internet of Things. Other terms such as telemental health, e-mental health, and connected mental health could also be used to describe related concepts [8].

### Goal of This Study

Telepsychiatry is a broad concept, and it has many applications. This results in heterogeneity in the literature. One key aspect of telepsychiatry is its use by physicians for various aspects of depression care, including assessment, evaluation, diagnosis, management, and follow-up. This review therefore aims to evaluate the extent and nature of the existing literature on telemedicine for the care of depression by physicians. This review also aims to examine the effects and perceptions of virtual care and how it compares to traditional in-person care.

## Methods

### Theoretical Framework

This scoping review was conducted by following the Arksey and O’Malley framework as well as the PRISMA-ScR (Preferred Reporting Items for Systematic Reviews and Meta-Analyses Extension for Scoping Reviews) guidelines. The five methodological stages were the identification of the research question, the identification of relevant articles, the study selection, the data extraction, and the collating, summarizing, and reporting of the results [9].

### Identification of the Research Question

As described above, this review’s main objectives are to map the literature on telemedicine in the diagnosis and management of depression by physicians and to examine the effects of telemedicine and how it is perceived. In other words, this review examines the virtual care of patients with depression by physicians. The identification of this research question is closely related to the burdens of depression and the potential of innovative psychiatric care.

### Identification of Relevant Articles

A systematic literature search was performed in MEDLINE, Cochrane Database of Systematic Reviews and PsycARTICLES. These databases were chosen based on their relevance and specificity for peer-reviewed articles on medical and psychiatric topics. No search for gray literature was performed because the scope of this review did not extend to articles that had not been peer-reviewed. The list of keywords used was developed through a preliminary search on PubMed, and the keywords were selected based on their sensitivity and specificity for relevant articles. The search terms used for the database searches in this review are listed in Textbox 1. The literature search was performed on October 11, 2020, and it yielded 4782 articles.

**Search string with the keywords used for the database searches.**

(*virtual* OR *telemedicine* OR teleconsultation* OR *telehealth* OR phone* OR webcam* OR *telepsychiatry*) AND (depress*)

### Study Selection

Following removal of all 532 duplicates, the remaining 4250 articles were screened based on the eligibility criteria detailed in Textbox 2. Articles were initially screened based on their title and abstract, and full text articles were obtained when more information was needed for screening. The final count of studies included in this review is 28.

**Eligibility criteria for study selection.**

**Inclusion criteria**

- Directly related to telemedicine
- Directly related to the evaluation or treatment of depression
- Care provided by physicians (eg, general practitioners, psychiatrists)

**Exclusion criteria**

- Mobile health apps as the primary focus
- Texting and emails as the primary focus
- Psychotherapy as the main intervention (eg, articles about web-based cognitive behavioral therapy)
- Psychotherapy or care provided by health care professionals other than physicians
- Depressive symptomatology from bipolar disorder, anxiety disorder, or schizoaffective disorder
- Depressive symptomatology as an outcome of telemedicine for diseases other than depression (eg, multiple sclerosis, heart failure, cognitive impairment)
- Data from patients with depression mixed with data of patients without depression (eg, telepsychiatry in general, anxiety and depression)
- Not peer-reviewed
- Published research protocols that had not yet been completed
- Conference proceedings
- Supported neither by empirical data nor by a formal literature review (eg, editorials)
- Published in a language other than English or French
- Full article could not be obtained

### Data Extraction

An Excel file (Microsoft Corporation) was used to chart the data from all included articles. Throughout the charting process, the extraction grid was iteratively revised to refine its components.

### Collating, Summarizing, and Reporting the Results

Results regarding the study characteristics for both the included review and nonreview articles are reported below. However, analysis of the outcomes and results of the included articles was limited to nonreview articles because the included reviews had high heterogeneity and their aims tended to differ significantly from the scope of this scoping review. In-depth analysis of outcomes and results was also limited to the scope of this review. Tables were used to report relevant data comprehensively. Figures were produced using Excel.

## Results

### Selection Process

**Figure 1:**
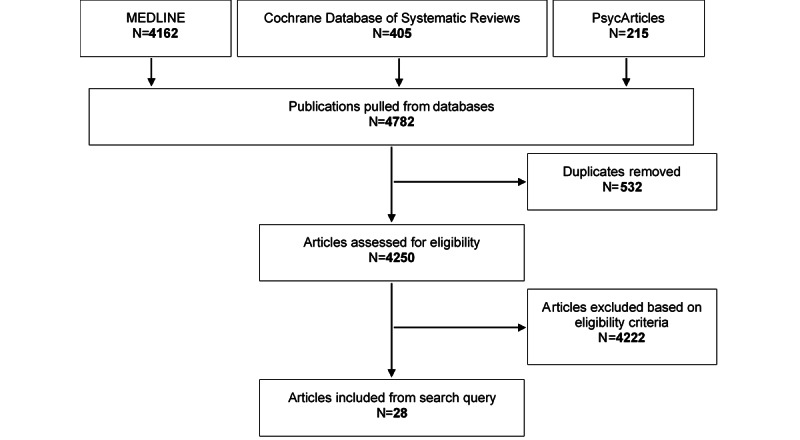
Study selection flow diagram.

A total of 28 articles were included in this review. The study selection process is detailed in Figure 1 [10-37].

### Characteristics of the Included Articles

Although all the articles were published in peer-reviewed journals, an absence of conflicts of interest was clearly and explicitly declared in only a small majority (17/28).

**Figure 2:**
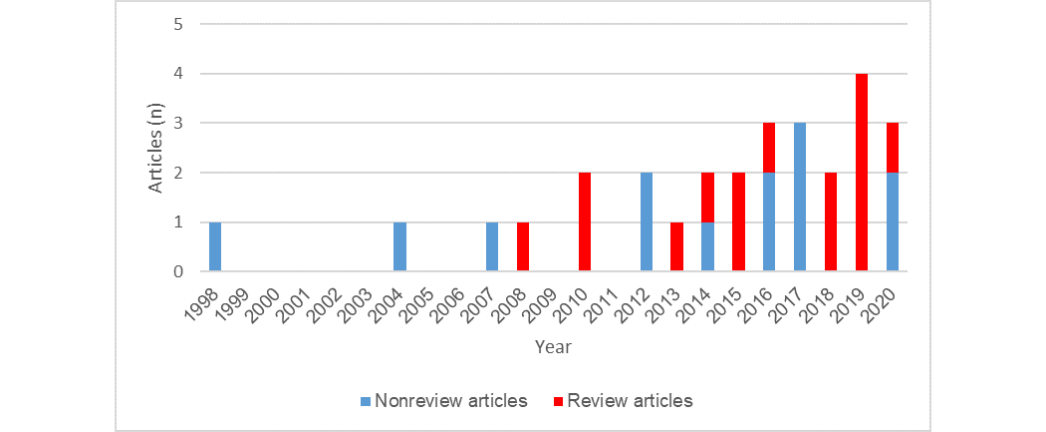
Histogram of the numbers of review and nonreview articles published per year.

All included papers were published between 1998 and 2020. The great majority had been published since 2010 (24/28), and more than half had been published since 2015 (17/28). The publication years of the papers are illustrated in Figure 2.

A total of 13 studies were conducted using a nonreview methodology, while 15 articles were reviews. Most nonreview studies (9/13) compared a virtual intervention group and a nonvirtual group, and almost all of these comparative studies (8/9) included a randomization process.

Most nonreview studies were conducted in the United States. Locations represented in nonreview articles are displayed in Table 1. Another notable characteristic of the populations of these studies was their size, which ranged from 1 to 309 patients. The average study population size was 135 patients. The intervention in almost all these studies included a psychiatrist (12/13) instead of a primary care provider or as well as one. Collaborative care was part of most interventions (7/13). A nonvirtual comparison group was included in nine studies. Characteristics of the populations, virtual interventions, and nonvirtual comparisons of the nonreview studies are detailed further in Table 2 [10-22].

**Table 1:** Study locations with the associated numbers of nonreview articles (n=13).

Location	Nonreview articles, n (%)
United States	10 (76.9)
Europe	1 (7.7)
Brazil	1 (7.7)
Columbia	1 (7.7)

**Table 2:** Population, virtual intervention, nonvirtual comparison, outcomes and results relevant to the scope of this review for the included nonreview articles.

Authors (year)	Population	Intervention (virtual)	Comparison (nonvirtual)	Outcomes and results
Amirsadri et al (2017) [15]	1 patient; United States; comorbid schizophrenia	Videoconferencing with a telepsychiatrist; home visits by a social worker and a nurse	None	Diagnosis: Detecting undiagnosed depression. Treatment: Development of a treatment plan. Function: Improved. Quality of life: Improved. Longitudinal history: Discovery of trauma history. Patient satisfaction: Very happy with the service. Wanted it to be available for others. Caregiver satisfaction: Very convenient. Good care.
Barrera-Valencia et al (2017) [12]	106 patients; Colombia; inmates	2 interventions: asynchronous care by the telepsychiatrist, who receives clinical information from the general practitioner’s evaluation; synchronous care by the telepsychiatrist through videoconferencing	None	Depression severity: Significant improvement in the Hamilton Depression Rating Scale scores for both telepsychiatry modalities. Higher effectiveness for the asynchronous modality. Consultation time: No difference between the two telepsychiatry modalities. Consultation costs: Higher for the synchronous modality. Cost-effectiveness: Higher for the asynchronous modality. Patient satisfaction: A dependent variable for the prediction of the cost of care.
Choi Yoo et al (2014) [11]	309 patients; United States; patients with cancer	INCPAD^a^ trial; centralized telecare management; automated home-based symptom monitoring; collaborative care by an oncologist, a nurse depression-pain care manager, and a supervising psychiatrist	Treatment as usual; screening results provided to their oncologist	DFDs^b^: 227.38 for the intervention group during the 12-month follow-up compared with 167.08 for the usual care group. This is an increase of 60.30 DFDs (*P*<.01) compared to the usual-care group. QALYs^c^: With 0.2 to 0.4 QALYs per additional DFD, there was a gain of between 0.033 and 0.066 QALYs. By other metrics, the intervention resulted in a gain of between 0.013 and 0.088 QALYs. Physician time cost^d^: $43,226 total and $281 per patient. Nurse care manager costs: $61,906 total and $402 per patient. Automated monitoring system cost: $506 per patient. Sum of the physician, nurse care manager and monitoring cost: $1189 per patient. Post–start-up automated monitoring maintenance cost over the 3 years of the trial: $20,000 total and $813 per patient. Cost per DFD: Between $19.72 and $26.95. Cost per QALY: $10,826 to $73,286. Post–start-up incremental cost per DFD gained: $13.48. Post–start-up incremental cost per QALY gained: Between $7564 and $51,199.
Chong and Moreno (2012) [13]	167 patients; United States; Hispanic participants; low-income, uninsured, and underinsured	Videoconferencing with a telepsychiatrist	Treatment as usual; primary care setting	Appointment keeping: Same for both groups. Visit satisfaction: Higher for the intervention group Patient rating of the working alliance with their provider: Higher for the intervention group Use of antidepressant medication: Higher for the intervention group. Depression severity: Both groups showed a decrease in symptoms. Depression severity improvement rate: Higher for the intervention group. Number of days in which their symptoms rendered them less productive or worse, unable to carry out their normal responsibilities: Decrease for both groups. Whether the project helped or made them better: 84% of both groups indicated that it did help them or make them better. Satisfaction with provided support: 50% of both groups indicated that the project and staff provided much-appreciated support. Satisfaction with session length: 12% of the intervention group patients wanted longer sessions. Satisfaction with session quantity: 12% of the comparison group wanted more sessions. Satisfaction with the webcam sessions: 22% of the intervention group patients mentioned that they liked the webcam sessions. Need for time to adapt: 14% of the intervention group patients reported that they needed some time to adapt. Willingness to pay for mental health services: High for both groups. Depression care satisfaction rating: High for both groups. Satisfaction with the randomized assignment they received: High for both groups.
Christensen et al (2020) [22]	199 patients; 11 European countries; mild to moderate depression.	MasterMind program; videoconferencing facilitating collaborative care interventions; telepsychiatrists, general practitioners, and other health care professionals	None	Patient satisfaction: High. Scores varied significantly between regions, but there was no correlation with age and gender.
Emery-Tiburcio et al (2017) [16]	131 patients; United States; aged ≥60 years	BRIGHTEN^e^ program; telepsychiatrists and other health care professionals	None	Depression severity: Significant improvements in Geriatric Depression Scale rating and 12-Item Short Form Survey Mental Health Composite at 6-month follow-up. Equal benefit from the program for individuals with different ethnic, educational, and income characteristics. 12-Item Short Form Survey Physical Health Composite: No differences observed at 6-month follow-up.
Hilty et al (2007) [10]	121 patients; United States; rural	Intensive disease management module; videoconferencing with a telepsychiatrist; training for the primary care physician	Usual care; disease management module	Depression severity: Significant reduction at 3, 6, and 12 months for both groups, with no significant difference between the groups. According to a post-hoc analysis, the score for one depression subscale score was nearly significantly higher in the intervention group when those in the comparison group with an initial telepsychiatric consultation were removed from the analysis. There was a relationship between depression and health functioning scores. No relationship was found between depression scores and satisfaction. Health functioning: No significant change or significant difference between groups. There was a relationship between depression and health functioning scores. No relationship was found between satisfaction and health functioning. Patient satisfaction: Significantly higher in the intervention group at 6 and 12 months. No relationship between depression scores and satisfaction; no relationship between satisfaction and health functioning. Study retention: Significantly higher in the intervention group; higher in older patients. Comorbid somatization, phobia, and anxiety: Significantly higher improvement in the intervention group
Hungerbuehler et al (2016) [14]	107 patients; Brazil; 3 hours of mean traveling time to psychiatric hospital	Videoconferencing with a telepsychiatrist; significantly higher baseline depression severity	Treatment as usual; in-person consultations with a psychiatrist	Depression severity: Significant decrease in severity for both groups. No significant difference in severity between groups at 6 and 12 months. Significant interaction between treatment and time regarding severity. Medication: Most of the participants continued taking antidepressants at 6 and 12 months within the recommended dosages, often combined with sedatives. No significant difference between groups for the type and dosage of medication at 6 and 12 months. Low adherence in both groups. No significant difference in adherence between groups. Treatment adherence validated by the number of missed appointments and dropouts: Significantly more dropouts in the nonvirtual group at 6 months, but no significant difference at 12 months. More missed appointments in the nonvirtual group. Patient satisfaction: No significant difference between groups at 6 and 12 months. A significant increase was observed during the first 6 months and then remained stable until the end of the study. No significant changes over the entire study period. No significant interaction between group assignment and time. Working alliance: Significant increase over 12 months for both groups. No significant difference between groups
Moreno et al (2012) [20]	167 patients; United States; Hispanic	Videoconferencing with a telepsychiatrist	Treatment as usual; community health center	Depression symptoms: Significant reduction in both groups. Significantly higher reduction in the virtual group. Significant effect of time. Depression response (50% or greater decrease in severity ratings): More than half of the study population. No significant difference between groups, but tendency for higher rate in the virtual group. Depression remission (75% or greater decrease in severity ratings): Less than half of the study population. No significant difference between groups, but tendency for a higher rate in the virtual group. Quality of life: Significant increase for both groups. Significantly higher increase in the virtual group. Significant effect of time. Health-related functional ability: Significant increase for both groups. Significantly higher increase in the virtual group. Significant effect of time.
Norden et al (2020) [21]	114 visits; United States; Stanford’s Accountable Care Organization’s patient population (younger, healthier, more tech-savvy)	Stanford ClickWell Care, a novel virtual primary care clinic; videoconferencing or telephone visits with a primary care provider	In-person visits at the Stanford ClickWell Care clinic with a primary care provider	Number of visits: Significantly higher in the virtual setting than in-person. Number of labs ordered: No significant difference between groups. Number of images ordered: No significant difference between groups.
Ruskin et al (1998) [17]	30 patients; United States; psychiatric inpatients	1 in-person consultation with a psychiatrist; 1 videoconferencing consultation with a telepsychiatrist	2 in-person consultations with a psychiatrist	Interrater reliability for results from the Structured Clinical Interview for DSM-III-R^f^: High for both groups. No significant difference between groups. Patient satisfaction: High for both groups. No significant difference between groups. For the intervention group, “Overall, which did you prefer?”: Most preferred the in-person consultation. For the intervention group, “Would you rather have a video examination with a psychiatrist or an in-person examination by a general practitioner who might know a little less about psychiatry?”: Most preferred the virtual consultation. For the intervention group, “If you lived two hours away from the hospital, would you rather travel to the hospital to see the psychiatrist in person or go to a place close to your home and see the psychiatrist by video?”: Most preferred the virtual consultation.
Ruskin et al (2004) [19]	119 patients; United States; veterans	Videoconferencing with a telepsychiatrist	In-person consultations with a psychiatrist	Depression severity: Significant reduction in both groups. No significant difference between groups. Depression response (50% improvement from the first to the last visit): Similar rate in both groups. Depression remission (17-item Hamilton Depression Rating Scale score ≤7): Similar rate in both groups. Treatment adherence in terms of dropout rates, time course of dropouts, number of session appointments kept, and pill counts: No significant difference between groups. Patient satisfaction: High for both groups. No significant difference between groups. Psychiatrist satisfaction: High for both modalities of care. Significantly higher for nonvirtual care. Resource consumption or “cost effects” through per-session cost with or without factoring psychiatrist travel time and total Veterans Affairs health care resource consumption: $86.16 for a telepsychiatry session and $63.25 for an in-person session. Equal cost if the psychiatrist had to travel 22 miles to the clinic. Modality of care was not associated with significantly different consumption of health care.
Yeung et al (2016) [18]	190 patients; United States; monolingual Chinese American immigrants	T-CSCT^g^; T-CSCT involving culturally sensitive psychiatric assessment, and collaborative care; videoconferencing with a bilingual telepsychiatrist; primary care provider, bilingual telepsychiatrist, and bilingual care manager	Treatment as usual; in-person consultations with a primary care provider; a single videoconferencing evaluation with a telepsychiatrist; treatment recommendations from the telepsychiatrist	Depression severity: Significantly higher reduction for the intervention group. Depression response rate (Hamilton Depression Rating Scale score improvement of ≥50%): Significantly higher for the intervention group. Depression remission rate (Hamilton Depression Rating Scale score ≤7): Significantly higher for the intervention group. Quality of life: No significant difference between groups.

^a^INCPAD: Indiana Cancer Pain and Depression.

^b^DFD: depression-free day.

^c^QALY: quality-adjusted life year.

^d^All monetary values are reported in US dollars.

^e^BRIGHTEN: Bridging Resources of an Interdisciplinary Geriatric Health Team via Electronic Networking.

^f^DSM-III-R: Diagnostic and Statistical Manual of Mental Disorders (Third Edition, Revised).

^g^T-CSCT: Telepsychiatry-based culturally sensitive collaborative treatment.

### Main Results for Nonreview Articles

Of the 13 nonreview papers, 11 included results regarding depression variables, 8 included results with regard to perceptions, and 11 included results regarding other variables. Outcomes and results relevant to the scope of this review for the included nonreview articles are detailed in Table 2 [10-22].

Regarding depression outcomes and variables, all 11 studies resulted in improvement in depression with the use of virtual care, and studies that compared the virtual intervention group with a nonvirtual comparison group either obtained equivalent improvement or better results with virtual care. Studied depression outcomes and variables include depression severity, severity improvement rate, depression-free days (DFDs), response rate, remission rate, treatment aspects such as adherence, and depression interrater reliability for diagnosis.

Perceptions regarding virtual care were favorable to virtual care or equivalent to in-person care according to almost every metric studied. Examined perceptions include patient satisfaction, caregiver satisfaction, psychiatrist satisfaction, working alliance, need for time to adapt, willingness to pay for mental health services, and care preferences.

Other variables were significantly improved with virtual care. Studied variables included quality of life, quality-adjusted life years (QALYs), functioning, appointment keeping, number of visits, number of complementary tests ordered, physical health, and comorbid somatization, phobia, and anxiety.

It was also proven that telepsychiatry can be more cost-effective than in-person psychiatry. Examined costs and related variables include consultation time, consultation cost, cost-effectiveness, per-session costs, physician time cost, nurse care manager cost, system cost, system maintenance costs, cost per DFD or QALY, and post–start-up incremental cost per DFD or QALY gained.

## Discussion

### Overview of the Literature

This review aimed to map the literature on use of telepsychiatry to treat depression by physicians as well as to examine the effects and perceptions regarding telepsychiatry and how it compares to traditional in-person care. A total of 28 articles were included [10-37], and all 13 nonreview articles were analyzed further [10-22]. The generalizability of the findings may be modulated by the fact that most studies were recent, but it may be limited for primary care populations and countries other than the United States. Although quality appraisal of the studies was not part of the methodology of this study, bias risk may be modulated by the high proportion of randomized studies, the low proportion of clear and explicit declared absence of conflicts of interest, and the average study size, which may arguably be considered sufficiently small or large. The analyzed articles contained measured outcomes related to depression variables, perceptions, and other variables. All applicable studies resulted in improvement in depression with the use of telepsychiatry, which was always measured to be equivalent or better for virtual care compared with in-person psychiatry. Patient satisfaction and other perceptions were examined, and telepsychiatry again performed at least as well as in-person care according to almost every metric studied. Quality of life, functioning, and similar variables were significantly improved with virtual care. Telepsychiatry also tended to perform better than in-person care. Cultural sensitivity and collaborative care were also part of some studied telemedicine programs.

### Gaps in the Literature

Additional research should be conducted on themes already covered in the articles included in this review. These themes include depression outcomes, perceptions such as patient satisfaction, cost-efficiency, and other outcomes. However, stakeholders would benefit from larger study populations, more studies conducted outside the United States, and more studies about general practitioners, as well as fewer potential conflicts of interest and more transparency.

Research should also be conducted on aspects of telepsychiatric care of patients with depression that are not covered as extensively in the included articles. These aspects include effects on suicide risk, suicidal ideation incidence, evolution of comorbid diseases, health behaviors, societal productivity, work productivity and absenteeism, personal and household income, marital outcomes, and parenthood outcomes, as well as the well-being of caregivers and relatives. Many perceptions could be studied, including those of patients, physicians, health care professionals, caregivers, and relatives. Studies should also be conducted regarding medical student training in telepsychiatry [5].

Research themes could be explored through various methodologies, including systematic reviews and randomized clinical trials.

### Limitations

The results of this review are subject to limitations. As definitions of telepsychiatry may differ, the scope of this review might also be considered too narrow in various ways, such as its focus on physicians, its exclusion of psychotherapy as the main intervention, its exclusion of patients with anxiety disorders, and other eligibility criteria that may be considered limited or firm. The chosen search terms may have further influenced the identification of relevant articles in this direction or may have hindered the identification of relevant articles such as studies about telephone medicine, asynchronous telemedicine, pediatricians, or special patient populations. The selected databases may have also influenced the results of this review; however, this decision was supported by the characteristics of the selected databases as well as the preliminary search. Arguably, other databases could have been considered. Similarly, not searching the gray literature may have influenced the results; however, it was decided that this review would be limited to peer-reviewed articles. Backward and forward reference searching has not been conducted. Including other professions may have better covered some research themes, such as quality of work life, quality of personal life, health behaviors, and other aspects. Some may also disapprove of the use of the PRISMA-ScR guidelines or the Arksey and O’Malley framework [9]. The choice to include a case study may be criticized, as the study population consists of only 1 patient [15]. Finally, the generalizability of the findings may be limited for certain locations, medical specialties, patient subsets, and other clinical environment characteristics not represented among the included articles.

### Conclusions

This review examines the literature on telemedicine in the care of depression by physicians, as well as its related effects and perceptions. More research is recommended to fully understand the current and potential roles of telepsychiatry when used by physicians caring for patients with depression. This research should examine various outcomes, including depression variables such as symptom severity and suicidal risk, perceptions such as stakeholder satisfaction and working alliance, health care outcomes such as cost-effectiveness, and other outcomes such as quality of life and work productivity. Studies should be conducted in various clinical contexts, such as urban or rural primary care, and in developing countries.

This review suggests that telemedicine tends to be at least as effective for depression care compared with in-person care, and it may be more cost-effective. Patient satisfaction tends to be high and perceptions tend to be favorable.

As depression is a highly prevalent and burdensome disease, its toll on patients’ mental and physical health as well as its health care burden can probably be reduced by improving and implementing virtual psychiatric care of depression by physicians.

## Abbreviations

DFD: depression-free day
GMF-U: groupe de médecine de famille universitaire (university family medicine group)
PRISMA-ScR: Preferred Reporting Items for Systematic Reviews and Meta-Analyses Extension for Scoping Reviews
QALY: quality-adjusted life year

## References

[1] Kessler RC (2012). The costs of depression. Psychiatr Clin North Am.

[2] Möller HJ (2003). Suicide, suicidality and suicide prevention in affective disorders. Acta Psychiatr Scand Suppl.

[3] Greenberg PE, Fournier A, Sisitsky T, Pike CT, Kessler RC (2015). The economic burden of adults with major depressive disorder in the United States (2005 and 2010). J Clin Psychiatry.

[4] Guaiana G, Mastrangelo J, Hendrikx S, Barbui C (2021). A systematic review of the use of telepsychiatry in depression. Community Ment Health J.

[5] Echelard J, Méthot François, Nguyen H, Pomey M (2020). Medical student training in eHealth: scoping review. JMIR Med Educ.

[6] Unützer J, Katon W, Callahan CM, Williams JW, Hunkeler E, Harpole L, Hoffing M, Della PRD, Noël PH, Lin EHB, Areán PA, Hegel MT, Tang L, Belin TR, Oishi S, Langston C (2002). Collaborative care management of late-life depression in the primary care setting: a randomized controlled trial. JAMA.

[7] Yeung A, Chang D, Gresham RL, Nierenberg AA, Fava M (2004). Illness beliefs of depressed Chinese American patients in primary care. J Nerv Ment Dis.

[8] Drissi N, Ouhbi S, Janati Idrissi MA, Fernandez-Luque L, Ghogho M (2020). Connected mental health: systematic mapping study. J Med Internet Res.

[9] Arksey H, O'Malley L (2005). Scoping studies: towards a methodological framework. Int J Soc Res Methodol.

[10] Hilty DM, Marks S, Wegelin J, Callahan EJ, Nesbitt TS (2007). A randomized, controlled trial of disease management modules, including telepsychiatric care, for depression in rural primary care. Psychiatry (Edgmont).

[11] Choi Yoo SJ, Nyman JA, Cheville AL, Kroenke K (2014). Cost effectiveness of telecare management for pain and depression in patients with cancer: results from a randomized trial. Gen Hosp Psychiatry.

[12] Barrera-Valencia C, Benito-Devia AV, Vélez-Álvarez C, Figueroa-Barrera M, Franco-Idárraga SM (2017). Cost-effectiveness of synchronous vs. asynchronous telepsychiatry in prison inmates with depression. Article in Spanish. Rev Colomb Psiquiatr.

[13] Chong J, Moreno F (2012). Feasibility and acceptability of clinic-based telepsychiatry for low-income Hispanic primary care patients. Telemed J E Health.

[14] Hungerbuehler I, Valiengo L, Loch AA, Rössler Wulf, Gattaz WF (2016). Home-based psychiatric outpatient care through videoconferencing for depression: a randomized controlled follow-up trial. JMIR Ment Health.

[15] Amirsadri A, Burns J, Pizzuti A, Arfken CL (2017). Home-based telepsychiatry in US urban area. Case Rep Psychiatry.

[16] Emery-Tiburcio EE, Mack L, Lattie EG, Lusarreta M, Marquine M, Vail M, Golden R (2017). Managing depression among diverse older adults in primary care: the BRIGHTEN program. Clin Gerontol.

[17] Ruskin PE, Reed S, Kumar R, Kling MA, Siegel E, Rosen M, Hauser P (1998). Reliability and acceptability of psychiatric diagnosis via telecommunication and audiovisual technology. Psychiatr Serv.

[18] Yeung A, Martinson MA, Baer L, Chen J, Clain A, Williams A, Chang TE, Trinh NT, Alpert JE, Fava M (2016). The effectiveness of telepsychiatry-based culturally sensitive collaborative treatment for depressed Chinese American immigrants: a randomized controlled trial. J Clin Psychiatry.

[19] Ruskin PE, Silver-Aylaian M, Kling MA, Reed SA, Bradham DD, Hebel JR, Barrett D, Knowles F, Hauser P (2004). Treatment outcomes in depression: comparison of remote treatment through telepsychiatry to in-person treatment. Am J Psychiatry.

[20] Moreno FA, Chong J, Dumbauld J, Humke M, Byreddy S (2012). Use of standard Webcam and Internet equipment for telepsychiatry treatment of depression among underserved Hispanics. Psychiatr Serv.

[21] Norden JG, Wang JX, Desai SA, Cheung L (2020). Utilizing a novel unified healthcare model to compare practice patterns between telemedicine and in-person visits. Digit Health.

[22] Christensen LF, Gildberg FA, Sibbersen C, Skjoeth MM, Nielsen CT, Hansen JP (2020). Videoconferences and treatment of depression: satisfaction score correlated with number of sessions attended but not with age. Telemed J E Health.

[23] Dorstyn DS, Saniotis A, Sobhanian F (2013). A systematic review of telecounselling and its effectiveness in managing depression amongst minority ethnic communities. J Telemed Telecare.

[24] Hilty DM, Rabinowitz T, McCarron RM, Katzelnick DJ, Chang T, Bauer AM, Fortney J (2018). An update on telepsychiatry and how it can leverage collaborative, stepped, and integrated services to primary care. Psychosomatics.

[25] Kaonga NN, Morgan J (2019). Common themes and emerging trends for the use of technology to support mental health and psychosocial well-being in limited resource settings: A review of the literature. Psychiatry Res.

[26] Naslund JA, Mitchell LM, Joshi U, Nagda D, Lu C (2020). Economic evaluation and costs of telepsychiatry programmes: A systematic review. J Telemed Telecare.

[27] Olden M, Cukor J, Rizzo AS, Rothbaum B, Difede J (2010). House calls revisited: leveraging technology to overcome obstacles to veteran psychiatric care and improve treatment outcomes. Ann N Y Acad Sci.

[28] Jiménez-Molina Álvaro, Franco P, Martínez Vania, Martínez Pablo, Rojas G, Araya R (2019). Internet-based interventions for the prevention and treatment of mental disorders in Latin America: a scoping review. Front Psychiatry.

[29] Fletcher TL, Hogan JB, Keegan F, Davis ML, Wassef M, Day S, Lindsay JA (2018). Recent advances in delivering mental health treatment via video to home. Curr Psychiatry Rep.

[30] Hubley S, Lynch SB, Schneck C, Thomas M, Shore J (2016). Review of key telepsychiatry outcomes. World J Psychiatry.

[31] Lopez A, Schwenk S, Schneck CD, Griffin RJ, Mishkind MC (2019). Technology-based mental health treatment and the impact on the therapeutic alliance. Curr Psychiatry Rep.

[32] García-Lizana F, Muñoz-Mayorga I (2010). Telemedicine for depression: a systematic review. Perspect Psychiatr Care.

[33] Chan S, Parish M, Yellowlees P (2015). Telepsychiatry today. Curr Psychiatry Rep.

[34] Massoudi B, Holvast F, Bockting CLH, Burger H, Blanker MH (2019). The effectiveness and cost-effectiveness of e-health interventions for depression and anxiety in primary care: a systematic review and meta-analysis. J Affect Disord.

[35] Hailey D, Roine R, Ohinmaa A (2008). The effectiveness of telemental health applications: a review. Can J Psychiatry.

[36] Chakrabarti S (2015). Usefulness of telepsychiatry: a critical evaluation of videoconferencing-based approaches. World J Psychiatry.

[37] Boydell KM, Hodgins M, Pignatiello A, Teshima J, Edwards H, Willis D (2014). Using technology to deliver mental health services to children and youth: a scoping review. J Can Acad Child Adolesc Psychiatry.

